# Habitat and Burrow System Characteristics of the Blind Mole Rat *Spalax galili* in an Area of Supposed Sympatric Speciation

**DOI:** 10.1371/journal.pone.0133157

**Published:** 2015-07-20

**Authors:** Matěj Lövy, Jan Šklíba, Ema Hrouzková, Veronika Dvořáková, Eviatar Nevo, Radim Šumbera

**Affiliations:** 1 Department of Zoology, Faculty of Science, University of South Bohemia, České Budějovice, Czech Republic; 2 Institute of Evolution, University of Haifa, Haifa, Israel; University of Missouri Kansas City, UNITED STATES

## Abstract

A costly search for food in subterranean rodents resulted in various adaptations improving their foraging success under given ecological conditions. In *Spalax ehrenbergi* superspecies, adaptations to local ecological conditions can promote speciation, which was recently supposed to occur even in sympatry at sites where two soil types of contrasting characteristics abut each other. Quantitative description of ecological conditions in such a site has been, nevertheless, missing. We measured characteristics of food supply and soil within 16 home ranges of blind mole rats *Spalax galili* in an area subdivided into two parts formed by basaltic soil and pale rendzina. We also mapped nine complete mole rat burrow systems to compare burrowing patterns between the soil types. Basaltic soil had a higher food supply and was harder than rendzina even under higher moisture content and lower bulk density. Population density of mole rats was five-times lower in rendzina, possibly due to the lower food supply and higher cover of *Sarcopoterium* shrubs which seem to be avoided by mole rats. A combination of food supply and soil parameters probably influences burrowing patterns resulting in shorter and more complex burrow systems in basaltic soil.

## Introduction

Mammals adapted for subterranean life have to deal with high energetic expenses for burrowing [1–3]. This is particularly true for rodents feeding on underground plant storage organs, as they must constantly extend their burrow systems in order to find new food resources. Success of the subterranean life in rodents might be enabled by various adaptations that improve their foraging success and/or reduce the risk of starvation (reviewed in [4]). Some of these adaptations involve strategies, which can be adjusted to local ecological conditions. For example, food-storing can be optimized for the size and nature of the food items available [4, 5], whereas burrow length and branching pattern could be adjusted to density and spatial distribution of these resources [6, 7]. Due to various adaptations and strategies subterranean rodents can even occupy habitats where the food is relatively sparse ([8] and citations therein). The food supply characteristics however, are not the only parameters affecting foraging success of subterranean rodents. The access to food resources is largely mediated by physical characteristics of the soil through which the animals burrow in search of these resources [9].

Blind mole rats of genus *Spalax* (Spalacinae, Rodentia) are Eurasian, primarily East-Mediterranean, solitary rodents adapted to strictly subterranean life [10]. Throughout their distributional range they live in various soil types and climatic zones [11]. For the last several decades, spalacines have been in the centre of scientific attention mainly due to the fact that they represent an actively speciating taxon [10, 12, 13]. In Israel, four chromosomal species of *Spalax ehrenbergi* superspecies, *S*. *golani* (2n = 54), *S*. *galili* (2n = 52), *S*. *carmeli* (2n = 58) and *S*. *judaei* (2n = 60), occupying four ecologically different climatic regions have been distinguished [12]. Each of those species occupies a different climatic region with a different combination of humidity and temperature. It is therefore supposed that the speciation in spalacines is grounded in adaptations for local ecological conditions following the gradient of aridity [14, 15].

So far, speciation in Spalacinae has been assumed to be allopatric and/or peripatric [10, 12]. Recently, in a microsite sharply subdivided geologically, edaphically, and ecologically, the incidence of incipient sympatric ecological speciation in *S*. *galili* has been proposed by Hadid et al. [16]. The authors detected differences between two abutting populations from the microsite in their genetics, behaviour and physiology. Nevertheless, ecological conditions such as food supply and soil characteristics, which could possibly lead to local adaptations and cause genetic divergence between the two mole rat populations have never been described using quantitative parameters.

*S*. *galili* is distributed in the cool and humid Upper Galilee Mountains in northern Israel [12] where terra rossa is the main soil type [17] and Mediterranean semi-shrubs called “batha” predominate [18]. We measured ecological parameters within home ranges of 16 *S*. *galili* in the microsite sharply subdivided into two adjacent edaphically different areas. In nine out of the 16 individuals we also excavated and mapped complete burrow systems. The aims of the study were (1) to quantify food supply and soil characteristics in the two soil types of the study microsite; (2) to describe and compare mole rat burrow system architecture in rendzina and basaltic soil. We predicted that mole rats living in the area with higher food supply and/or less workable soil would have shorter, but more complex burrow systems. (3) To reveal characteristics of the two populations studied, such as body mass distribution, sex ratio, population density and timing of reproduction.

## Material and Methods

### Ethic statement

This field study did not involve endangered or protected species. All procedures involving wild-caught animals were performed in a humane manner and were approved by the Institutional Animal Care and Use Committee at the University of South Bohemia and the Ministry of Education, Youth and Sports (n. 7942/2010-30). Since the fieldwork was conducted outside the protected areas and blind mole rats are considered to be agricultural pests in Israel, no specific permission was required for this study. After the end of the study, animals whose burrow systems were excavated were transported alive to the Animal Facility, Institute of Evolution, University of Haifa for further research purposes. All other individuals (re)captured were released into their original burrow systems after the shortest possible time.

### Study locality

**Fig 1:**
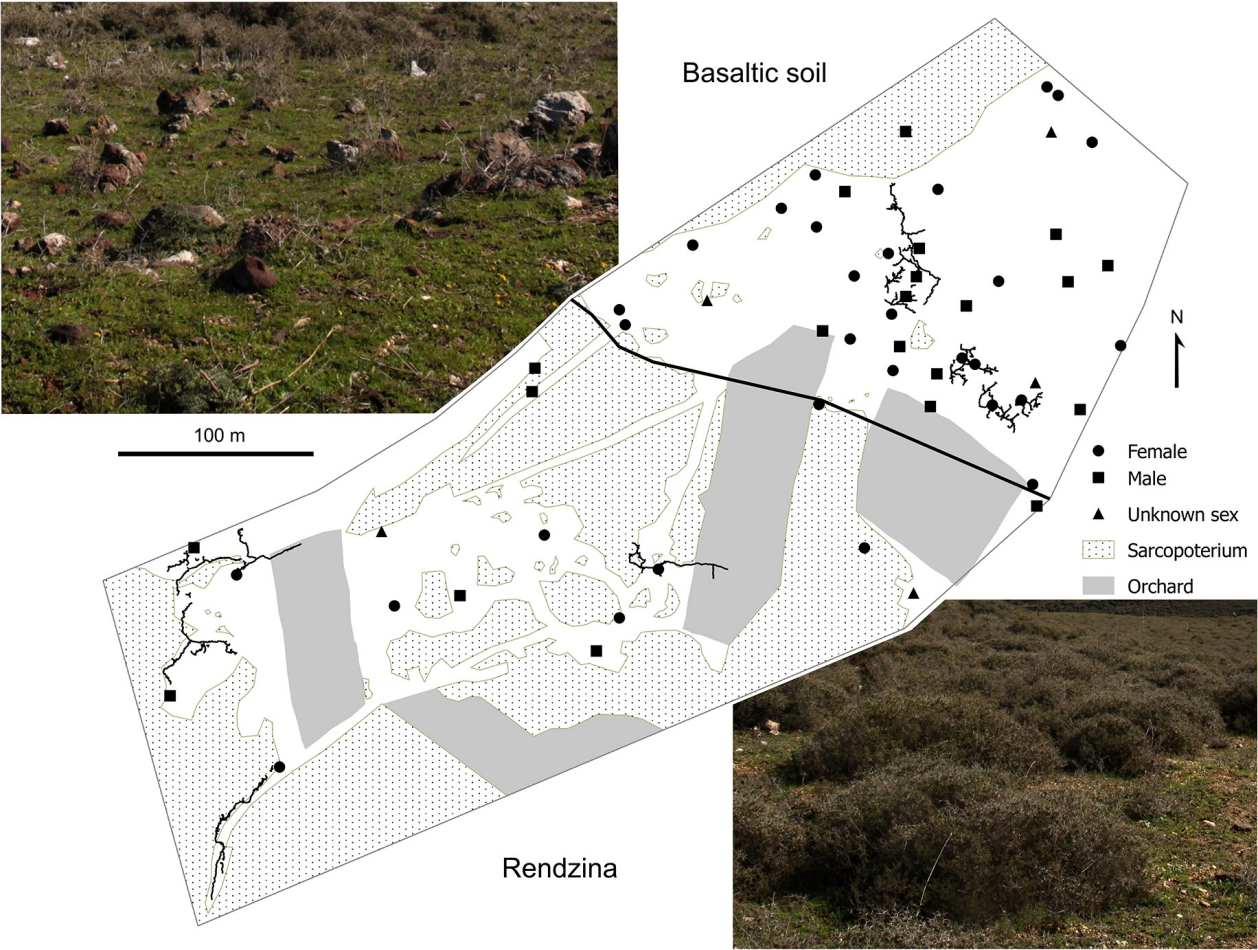
Map of the study microsite with all detected individuals of *S*. *galili*. A bold black line indicates the boundary between the rendzina and basaltic soil parts of the study microsite. Circles and squares represent captured females and males, respectively, and triangles indicate active territories from which animals had not been trapped. Burrow systems excavated are projected on the map. Photographs show typical vegetation types growing in rendzina and basaltic soil at the study microsite.

The study was conducted near Rihaniya in eastern Upper Galilee Mountains (33°02.5'N, 35°29.2'E, altitude 760 m). Predominating soil of the region is terra rossa, plus there are several islands of basaltic soil and pale rendzina [19]. The climate in Upper Galilee is Mediterranean, characterized by 714±163 mm of annual precipitation most of which (79±10%) falls in December–March (http://www.ncdc.noaa.gov/ghcnm/, accessed September 2014). The study microsite of approximately 12 ha covered by grazed Mediterranean batha vegetation with patches of olive orchards is partly on clay reddish-brown basaltic soil on basalt bedrock (4 ha) and partly on pale rendzina soil on white chalk/marl (8 ha) (see Fig 1, [19]). The basalt bedrock is of volcanic origin and is a part of the Dalton basalt plateau from the Late Pliocene 3.6–2.6 Mya [20]. In contrast, chalk and marl are sedimentary rocks and they formed much earlier during the Senonian Period 89–65 Mya [21]. The rendzina has higher CaCO_3_ content (80% in rendzina, 11–16% in basaltic soil), less organic matter content (more than 3-fold), lower C/N ratio (1.5 in rendzina, 6.2 in basaltic soil) and 2-fold lower water content in the summer [22]. The vegetation on the basalt is dominated by *Carlina hispanica*, whereas the dominant plant on the chalk is *Sarcopterium spinosum* (for a detailed species list, see [16] supporting information).

### Field work

Mole rats were captured using Hickman traps from November 2011 to March 2012 and they were weighed and sexed. 36 of the captured individuals (all>90 g) were radio-collared for the purpose of another study (for details on the radio-tracking methodology see [16]). The radio-tracking was carried out from 19. 1. to 6. 2. 2012. It started with 20 individuals and was successfully completed in 16 individuals (six from rendzina, nine from basaltic soil) [in prep.]. At the end of the radio-tracking study, we evenly placed five 0.5 m squares (mean distance between neighbouring squares in a burrow system was 13.5±6.1 m) along each burrow system of these 16 individuals (the burrow systems were roughly delimited by radio telemetry). In these squares, we quantified food supply and soil characteristics according to our previous studies on subterranean rodents (e.g. [4, 8, 23]). The squares were positioned to be less than 1 m from a tunnel or a cluster of mounds and were dug out to a depth of 20 cm. All aboveground green plant biomass (excluding *Sarcopoterium spinosum* and dry and woody tissues of other plants) and all underground storage organs of plants such as bulbs, corms, and swollen roots were collected and weighed with a laboratory scale (precision 0.1 g). On one side of each square, at a depth of 15 cm, we measured soil hardness using a hand penetrometer (06.06, Eijkelkamp, Agriresearch Equipment, Giesbeek, Netherlands). A 100 cm^3^ soil sample was then collected from the same depth from three squares (one in the middle and two on the peripheries of the home range) per individual. The soil sampling and soil hardness measurements took place two days after rain. The soil samples were subsequently oven-dried at 60°C to a constant weight and then sieved using a 2 mm mesh analytical sieve to separate coarse fragments.

After the end of radio-tracking, burrow systems of nine out of the 16 radio-tracked individuals (three males and three females from basaltic soil; one male and two females from rendzina) were excavated and mapped on graph paper. Tunnels backfilled with soil, but still identifiable, were also mapped. Within the burrow systems, we mapped positions of nests (chambers with bedding), toilets (chambers/blind ended tunnels with faeces) and food stores (chambers or blind tunnels with stored food). In all burrow systems, at approximately each meter during excavation, we measured the depth of the tunnels (96±65 measurements per burrow system, measured from its bottom to the ground surface) and the diameter, height and depth of nest chambers.

In order to study sex ratio, distribution of body masses, reproduction and spatial distribution of the individuals, we captured 28 extra individuals in addition to the 36 captured for the radio-tracking study. These 28 individuals included 11 mole rats captured in rendzina outside (<500 m) the microsite in order to increase the sample size. Within the microsite we mapped positions of remaining mole rats based on observations of fresh clusters of molehills. All individuals captured (all>80 g) occupied their own burrow systems and were therefore considered as adults (cf. [12, 24]). Through the study we collected information on mole rat reproduction, such as marks of pregnancy and lactation in captured females and presence of pups in their burrow systems.

### Data processing

In order to describe ecological conditions in the 16 mole rat home ranges, we quantified six parameters of food supply and four soil parameters based on the five sample squares per individual. The parameters of food supply were: underground storage organs' biomass per square meter, storage organs' density per square meter, green aboveground biomass per square meter, number of geophyte species per sample square, mass per storage organ, and Standardized Morisita index of dispersion of geophytes (*Ip*; [25]). The *Ip* was computed as a single number for rendzina and basaltic soil, respectively based on all 0.5 m squares of the respective soils pooled (50 in basaltic soil, 30 in rendzina). This index ranges from −1.0 (uniform distribution) to 1.0 (clumped distribution), with 95% confidence limits at −0.5 and 0.5. Perfectly random patterns give an index of zero. The parameters of soil were: soil hardness (cone resistance, N cm^-2^), soil moisture (percentage of fresh mass), soil density (g cm^-3^), and proportion of coarse fragments (percentage of dry mass).

Maps of excavated burrow systems were digitalized and analysed using the image processing software Quantum GIS [26]. To describe burrow architecture, we used the following indices: (1) burrow system area–calculated as the minimum convex polygon encompassing a burrow system, (2) complete burrow length, (3) length of main tunnel—the longest axial tunnel of the burrow system, (4) the number of branches per meter of the main tunnel, (5) Index of linearity (I_lin_; [23, 27]) and (6) fractal dimension (FD). The FD was calculated based on the box-counting method [28] using the programme ImageJ [29] with FracLac plugin [30], as described elsewhere [31]. The FD values were calculated to be comparable with previous studies on other subterranean rodents based on the manual box-counting method (for details see [23, 32]). Furthermore we calculated "unconstrained" FD (denoted as FD_u_), because the first method can substantially overestimate the true FD (for details see [23]).

### Statistical analyses

To compare food supply and soil parameters between basaltic soil and rendzina, we used nested design ANOVA. All the food supply and soil parameters had skewed distributions and they were therefore log transformed prior to all analyses. To compare the amount of stored food and mean tunnel depth between burrow systems from basaltic soil and rendzina, we used nonparametric Mann-Whitney U test. All the tests were performed with the R statistical package [33]. Interrelations between food supply and soil parameters measured for the nine mapped burrow systems and their relations to burrow system architecture were explored by principal component analysis (PCA) where parameters of burrow system architecture were passively projected to an ordination plot. Response variables entering the analysis (food supply and soil parameters) were centred and standardized to have zero mean and unite variance. The mean mass of underground storage organs was omitted from the PCA because it was not independent of the other two variables (density and biomass of underground storage organs). The burrow area was omitted from the analysis because it was highly correlated with total burrow length. PCA was performed with the software package CANOCO for Windows, version 4.52 [34]. To determine mole rats’ spatial distribution in basaltic soil and rendzina, we used the Average Nearest Neighbour Analysis and Spatial Statistics performed in ArcGIS 10.1. In this analysis, capture sites and places where the traps were plugged by the soil but the mole rats were not captured, represented positions of the animals. For each separate population, we calculated the nearest-neighbour index according to the following procedure. The distances between each animal and its nearest neighbour were measured. Subsequently, the index was calculated as the ratio of the mean observed nearest neighbour distance divided by the expected mean nearest neighbour distance. The expected mean nearest neighbour distance is based on a hypothetical random distribution with the same number of features covering the same total area. If the index is less than 1, the pattern exhibits clustering; if the index is greater than 1, the pattern exhibits dispersion. Finally, we tested whether the observed patterns deviated from the random pattern using *Z* statistics. *Z*-scores based on the Randomization Null Hypothesis [35] were calculated for each population and the statistical significance of *Z* was obtained from a probability table for the standard normal distribution (*Z* = 1.96, *p* = 0.05). If the *Z*-score is between -1.96 and 1.96, the pattern observed is likely to be random. To evaluate whether mole rats from rendzina avoided *Sarcopoterium* shrubs, we generated 17 random points within the rendzina part of the study area and performed χ^2^ test of the null hypothesis that mole rat presence is not related to the distribution of *Sarcopoterium* shrubs. To compare body mass of individuals of the same sex living in basaltic soil and rendzina, respectively, we used Student t-tests. To test whether sex ratios in both populations deviated from parity, we used χ^2^ tests. The Student t-tests and the χ^2^ tests were performed with the R statistical package [33]. Means ± standard deviations are given throughout the text.

## Results

### Soil and food supply characteristics

Most soil and food supply parameters significantly differed between basaltic soil and rendzina (see Table 1 for details on statistical tests). Basaltic soil was harder and moister, whereas rendzina was denser (Nested ANOVA, all *p*<0.0001). Green aboveground biomass of plants was higher in basaltic soil than in rendzina (Nested ANOVA, *p*<0.0001), as was also density of underground storage organs and number of geophyte species (Nested ANOVA, *p*<0.001 and *p*<0.0001, respectively). Underground storage organs of plants appeared to be smaller and their total biomass higher in basaltic soil, however these differences were not statistically significant (Table 1).

**Table 1:** Food supply and soil parameters of burrow systems of *Spalax galili* according to the soil type (Nested ANOVA).

Variable	Basaltic soil	Rendzina	F_1,14_	*p*
Green aboveground biomass (g m^-2^)	643±241	445±237	16.1	<0.0001
Biomass of underground storage organs (g m^-2^)	99.8±48.2	73.3±38.2	2.6	0.10
Density of underground storage organs (n m^-2^)	37.9±17.0	16.5±11.8	12.6	<0.001
Mean mass per storage organ (g)	2.3±2.1	3.3±3.3	2.7	0.11
Number of geophytes species (n sample^-1^)	2.8±1.2	1.8±0.9	21.3	<0.0001
Soil moisture (%)	24.3±1.7	19.9±2.1	63.8	<0.0001
Soil hardness (N cm^-2^)	33.8±11.4	17.3±9.6	44.1	<0.0001
Soil density (g cm^-3^)	1.19±0.08	1.33±0.12	40.3	<0.0001
Coarse fragments (%)	7.9±2.5	17.3±22.8	2.0	0.17

Altogether 603 underground storage organs of plants (roots and bulbs of grasses smaller than 0.1 g were not included) were found in the sample squares. *Ornithogalum lanceolatum*, *Ranunculus asiaticus* and *Eryngium creticum* were the most common plant species in basaltic soil comprising 28, 24, and 22% of all storage organs, respectively, whereas *E*. *creticum* (30%) predominated in rendzina, followed by *R*. *asiaticus* (18%) and one undetermined species (16%). Distribution of geophytes was clumped both in basaltic soil and rendzina, somewhat more in rendzina (*Ip* = 0.506 and 0.531, respectively).

### Burrow system architecture

**Fig 2:**
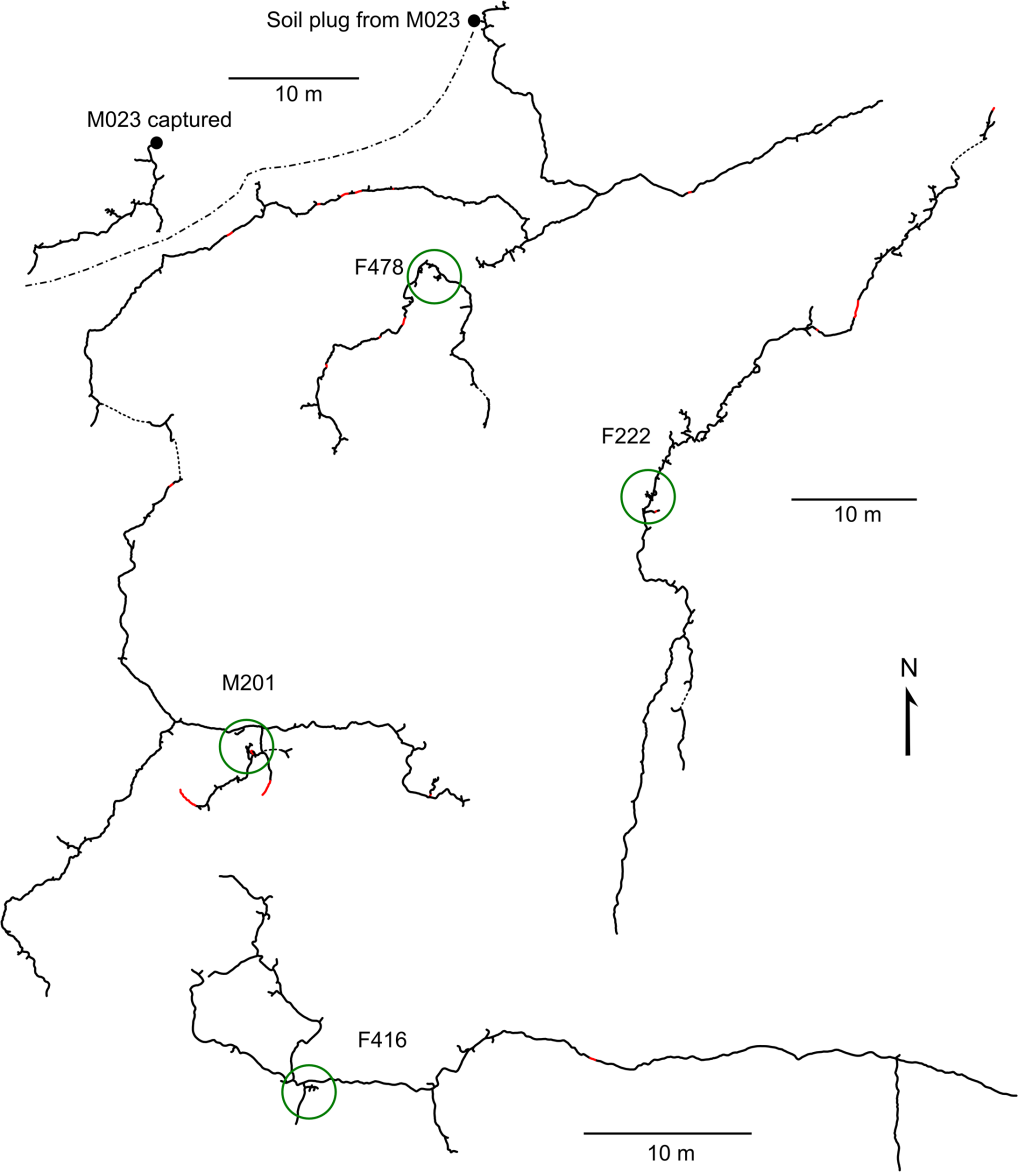
Burrow systems of *Spalax galili* in rendzina. Burrow systems of M201, F478 and M023 (partly excavated only) are depicted in real spatial configuration, for the true position of burrow systems of F222 and F416 see Fig 1. The burrow system of F478 was not included into analyses since the female was predated at the very beginning of the study. Red lines represent tunnels backfilled at a time of mapping, dashed lines are most probable connections not found. Green circles encompass nest sites. A dash-dot line indicates the expected boundary between the territories of males M201 and M023.

**Fig 3:**
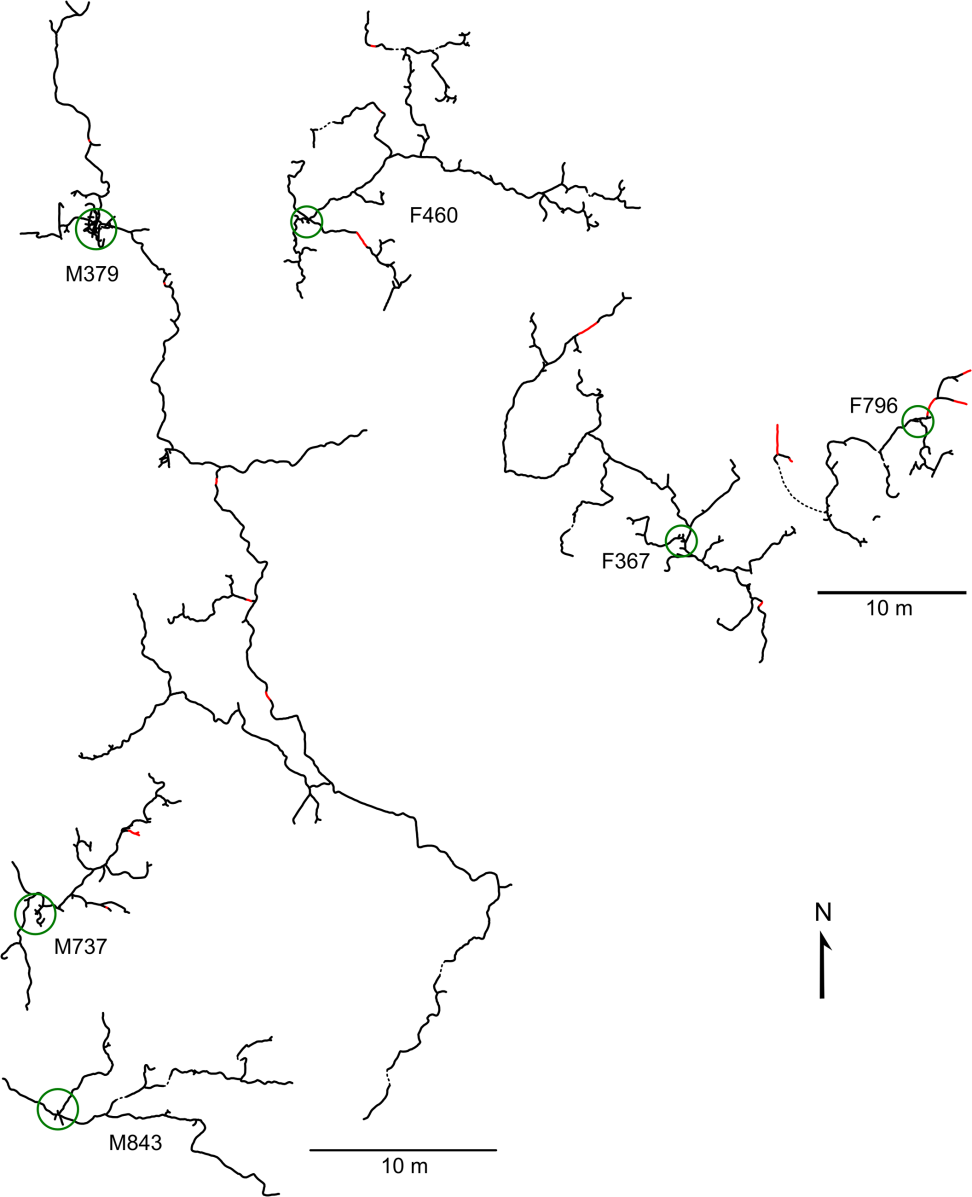
Burrow systems of *Spalax galili* in basaltic soil. Each triad of male burrow systems (M379, M737, M843) and female burrow systems (F460, F367, F796) is depicted in the real spatial configuration. Red lines represent tunnels backfilled at a time of mapping, dash lines are most probable connections not found. Green circles encompass nest sites.

For both soils combined, burrow systems of *S*. *galili* comprised 119±72 (46–275) m of tunnels covering an area of 647±929 (64–3023) m^2^ (MCP). They consisted of a main axial tunnel, lateral branches, nest chambers, and food stores (Table 2, Figs 2 and 3). Burrow systems were relatively shallow (usually 11–21 cm, Table 2), with the nests or blind tunnels close to the nests being the deepest parts. In five burrow systems (two from rendzina, three from basaltic soil), deep blind tunnels (>60 cm) were found near the nests. Detected backfilled tunnels comprised only 2.4±3.5 (0.3–11.3) % of the burrow systems but high soil moisture complicated their detection.

**Table 2:** Parameters of burrow systems of the blind mole rat *Spalax galili*. Stored food items were classified as aboveground and underground plant organs (M, male; F, female; I_lin_, index of linearity; I_con_, index of convolution; FD, fractal dimension; FD_u_, fractal dimension unconstrained).

Soil	Animal	Body mass (g)	Burrow area (m^2^)	Burrow length (% backfilled)	Main tunnel (m)	FD	FD_u_	branches/m of main tunnel	I_lin_	Mean tunnel depth (max)	Nest depth (cm)	Number of food stores: stored food (Aboveground+Belowground biomass) (g)
Basaltic soil	M379	210	870	172 (0.9)	89	1.25	1.06	0.22	1.33	22 (95)	40	1: 140+0
	M737	150	64	46 (2.1)	20	1.34	1.07	0.74	1.22	23 (70)	32	1: 2+16
	M843	119	101	57 (0.5)	27	1.36	1.06	0.30	1.17	17 (36)	32	0: 0
	F367	145	294	108 (1.8)	49	1.39	1.08	0.37	1.14	21 (30)	46	0: 0
	F460	137	312	118 (0.4)	39	1.42	1.10	0.39	1.11	19 (60)	36	1: 0
	F796	119	93	51 (11.3)	28	1.38	1.05	0.43	1.16	20 (46)	29	1: 0+11
	Mean		289±304	92±50 (3±4)	42±25	1.36±0.06	1.07±0.02	0.41±0.18	1.19±0.08	20±2 (56±24)	36±6	1: 24+5
Rendzina	M201	205	3328	303 (2.2)	154	1.35	1.07	0.25	1.16	16 (64)	53	5: 30+165
	F416	144	499	107 (0.3)	67	1.21	1.06	0.27	1.43	18 (45)	38	4: 6+326
	F222	126	566	140 (1.6)	96	1.18	1.05	0.45	1.83	22 (85)	70	4: 30+8
	Mean		1464±1614	183±86	106±36	1.25±0.07	1.06±0.01	0.32±0.09	1.47±0.28	19±3 (65±16)	54±13	4: 22+166

One currently used nest with bedding (an old nest was found in burrow systems of male M201 and female F367) was found in each burrow system (Table 2). The nest chambers had a diameter of 18±2 (16–24) cm, they were 16±4 (12–23) cm high and their floor was 42±13 (29–70) cm below the ground surface. Out of 11 nests excavated, nine had a single entrance and two had two entrances. Food stores (small chambers and short blind tunnels) were found close to the nests in seven out of nine burrow systems mapped (Table 2); female F416 had one additional food store 10 m from the nest. The food stores contained 122±126 (0–332) g of both aboveground (leaves, shoots etc.) and underground parts of plants (Table 2). The latter comprised mainly swollen roots (*E*. *creticum*, *Hordeum bulbosum*, Poaceae sp.) and bulbs (*O*. *lanceolatum* and several undetermined species). Mole rats from rendzina stored significantly more food than mole rats from basaltic soil (Table 2; Mann-Whitney U test, *p* = 0.026).

**Fig 4:**
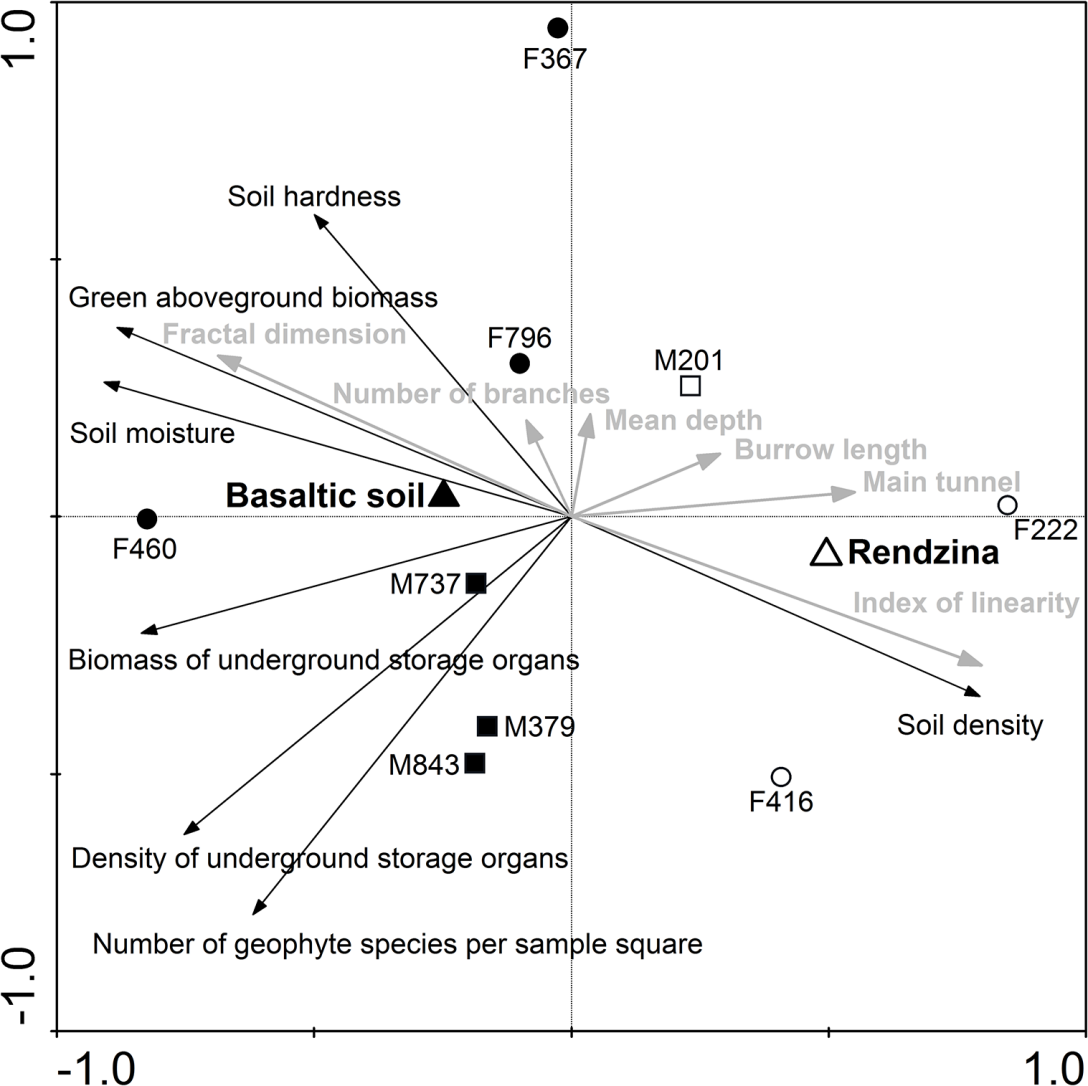
PCA ordination plot showing interrelationships between ecological characteristics (depicted in black) and passively projected burrow system parameters (in grey). Squares and circles represent males and females, respectively. Solid symbols represent individuals from basaltic soil, open symbols represent those from rendzina. Triangles are centroids for each habitat. The first ordination axis explains 52.5% of the data variability.

Inter-soil differences in the burrow system parameters and their relationships to soil and food supply characteristics are shown in Fig 4. The first ordination axis of the PCA ordination plot clearly differentiates between two soil types and explains 52.5% of the variability in food supply and soil parameters. Whereas in basaltic soil mole rats tended to have more complex and branched burrow systems characterised by a higher fractal dimension, burrow systems in rendzina tended to be longer and more linear (Fig 4, Table 2). In the ordination plot, the complexity of the burrow systems is associated mainly with higher aboveground biomass of plants and with higher soil moisture and hardness. In addition, the burrow length appears to be negatively correlated with biomass of underground storage organs of plants, their density and number of species per sample square (Fig 4). The depth of the burrow systems was similar in basaltic soil and rendzina (20±8 and 19±8 cm for basaltic soil and rendzina, respectively; Mann-Whitney U test, *p* = 0.38).

### Population structure

Altogether 56 active territories of *S*. *galili* (39 in basaltic soil, 17 in rendzina) were found at the study microsite. Population density was five-times higher in basaltic soil than in rendzina (10 and 2 individuals per ha, respectively; Fig 1). Individuals from both populations were randomly distributed within the respective parts of the study area (Nearest-neighbour Index = 1.13, 1.17; *Z*-score = 1.62, 1.33; *p* = 0.11, 0.18 for basaltic soil and rendzina, respectively). The mean nearest-neighbour distance between two individuals was twice greater in rendzina (39±21 [12–77] m) than in basaltic soil (18±9 [7–75] m). 14 out of 17 mole rat territories from rendzina were located outside the dense *S*. *spinosum* vegetation, which was significantly more than expected based on randomly generated points within the same study area (χ^2^ = 46.7, d.f. = 1, *p*<0.0001).

**Fig 5:**
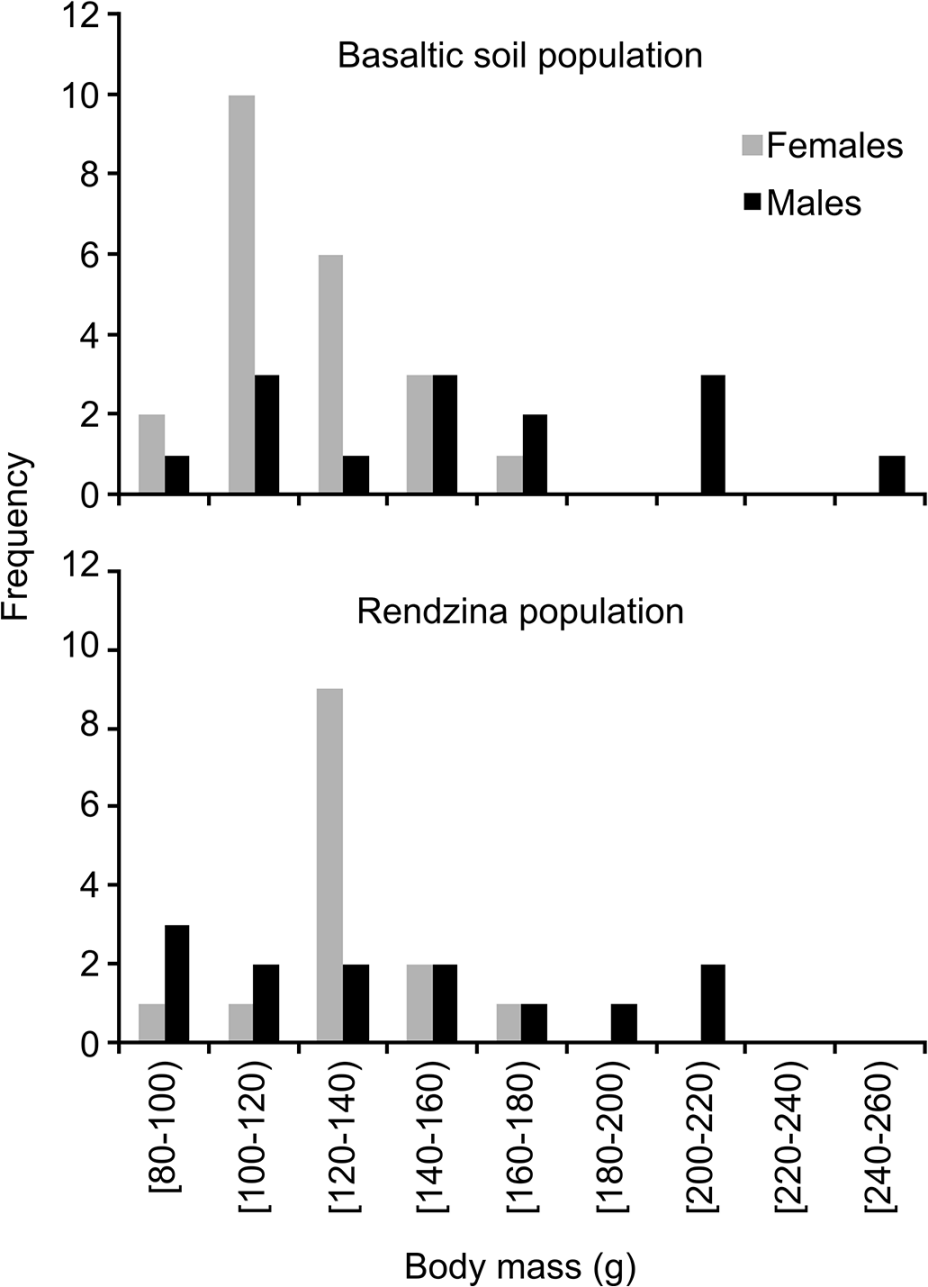
Histograms of body masses of basaltic soil and rendzina populations of *Spalax galili*.

Males were heavier (151±45, range 91–248 g, n = 36) than females (125±20, range 88–177 g, n = 27). There were no significant differences in body mass of males and females between the two populations (Student t-test; t = 1.29, -1.09; d.f. = 25, 31; *p* = 0.21, 0.28 for males and females, respectively). Body masses had a conspicuously narrow range in females (110–120 g in basaltic soil, 120–130 g in rendzina), whereas the body mass distribution in males was rather platykurtic in both populations (Fig 5). The sex ratio was female biased in the population inhabiting basaltic soil compared with that from rendzina, but it was not significantly different from parity in any of the soils (14 males and 23 females, 1:1.6, χ^2^ = 2.19, *p* = 0.14; 13 males and 14 females, 1:1.1, χ^2^ = 0.04, *p* = 0.84 for basaltic soil and rendzina, respectively).

Advanced pregnancies in three females and the presence of newborns at one nest were detected between 10. 2. and 10. 3. 2012. Marks of lactation in two females and signs of pre-weaning pups (presence of small diameter tunnels in female's F460 burrow systems) were detected during the first half of March. No differences between the two populations in the timing of reproduction were detected.

## Discussion

### Soil conditions

Both rendzina and basaltic soil inhabited by *S*. *galili* at the study microsite have developed in typical Mediterranean climate characterised by a strong annual alternation of a longer dry hot summer and a short cold and rainy winter [36]. Despite identical macroclimatic conditions, these soils differed significantly in all but one parameter (coarse fragments content, see Table 1) measured in our study.

One of the major factors affecting costs of burrowing in subterranean animals is soil water content since it directly influences density, cohesiveness, and shear strength of a given soil [1]. Many soils get very hard when dry and especially clay-rich soils typically shrink and harden during the long dry seasons in the Mediterranean region [36]. Very low summer moisture content in rendzina and basaltic soils were detected at the same microsite (3 and 8% respectively, [22]) suggesting that both soils may get very hard during the dry season. Importantly, this applies particularly to the basaltic soils because they usually contain a higher proportion of clay than rendzina (51±22% and 18% for basaltic soil and rendzina respectively, [37]).

More favourable conditions for burrowing arise in rainy season (winter) due to increased moisture [38]. Our findings indicate, however, that soils can differ markedly in their "workability" even in the rainy season (see Fig 4). A few days after a rainfall, basaltic soil was significantly harder than rendzina despite having a higher moisture content (see Table 1). Moreover, a combination of high moisture and clay content makes basaltic soil very sticky and plastic during the rainy season [39]. Mole rats living in basaltic soil therefore probably expend more energy on burrowing even in the rainy season. In addition, Shams et al. [40] found that mole rat burrows in “heavy” basaltic soil contained a dramatically lower level of O_2_ and higher level of CO_2_ than burrows in rendzina exactly at this microsite during the rainy season. This might possibly result in a higher need for constant reworking of the burrow systems in order to restore burrow atmosphere. We can therefore conclude that the basaltic soil is probably less workable and more stressful for blind mole rats than the rendzina year-round, not only during the dry season.

### Food supply characteristics

The diet of strictly subterranean rodents consists mainly of underground storage organs of plants [10], although, some of these rodents also consume aboveground biomass (see below). In our study, rendzina and basaltic soil differed in most food supply parameters, with basaltic soil offering higher density of underground storage organs of plants and green aboveground biomass. In this respect, mole rats burrowing in rendzina might be exposed to more stressful conditions, as reflected for example, in their lower resting metabolic rate compared with their basaltic-soil counterparts [16].

Irrespective of the soil type, biomass of underground storage organs of plants were among the lowest detected for a habitat of a subterranean rodent, including eusocial species of African mole-rats (see [8]). The only geophyte with large underground storage organs known to be consumed by mole rats (but not detected in the sampling squares) was *Asphodelus aestivus* which grows sparsely scattered only in rendzina. This raises the question how do blind mole rats persist in such a hostile environment? One explanation would be that spalacids feed partly on aboveground vegetation. Our regular observations of mole rats pulling a whole plant into their burrows and frequent occurrence of fresh leaves and stalks in their food caches support this hypothesis (see also [41, 42]).

Feeding upon green parts of plants might also be important for another reason; in a seasonal climate, nutrient-rich underground storage organs of some geophytes can be depleted of most resources due to a rapid leaf growth at the beginning of the flowering season [43]. We found a lot of wizened bulbs/tubers of plant species such as *Iris histrio* and *Ranunculus asiaticus* during the rainy season, indicating a decrease in their underground biomass. We propose that an opportunistic diet containing aboveground vegetation could be a suitable foraging strategy in blind mole rats enabling them (1) to preclude starvation in geophyte-poor patches/areas and (2) to compensate for the seasonal variability in the quality of underground plant storage organs.

Food storing is a common phenomenon in most subterranean rodents [10, 44]. The amount of stored food may reflect either the food availability in a given habitat or the necessity to store food in less productive habitats. Our finding that mole rats stored more food in rendzina, i.e. in soil with lower food supply, supports the latter hypothesis. In the solitary African mole-rat *Heliophobius argenteociereus*, the amount of stored food was positively correlated with food supply [4] and it has been suggested that its survival during the advanced dry season is conditioned by active searching for food, rather than storing a large amount of food. In Israeli spalacines, however, food reserves gathered during the winter are believed to suffice for the forthcoming long dry season [45]. Only small food reserves found in our study indicate that even the blind mole rats probably harvest food year-round. The role of food storing for the blind mole rat survival should be studied under more stressful conditions, such as in the advanced dry season with hard soil and reduced aboveground vegetation.

One of the most striking differences between the two parts of the study microsite was a higher abundance of *S*. *spinosum* bushes in rendzina compared with basaltic soil (see Fig 1). This dwarf-shrub occurs mainly on phosphorus-poor calcareous soils (such as rendzina in our study) and its seedlings hardly survive the moisture stress caused by cracking of the clayey soil during the dry season (such as in basaltic soil in our study) [46]. In addition, density and species richness of other vegetation such as forbs and grasses—the main food of spalacids–are dramatically reduced under this shrub [47]. As we have shown, sites with *Sarcopoterium* cover are avoided by mole rats. Thus, mainly *Sarcopoterium*-free gaps covered by grassy or herbaceous vegetation seem to provide suitable sites for mole rats living in rendzina. Since mole rat home ranges were mainly located in these gaps (Fig 1), we suppose that rendzina as a whole, offers in fact, even less biomass of food for mole rats than we assessed based on the food supply within mole rat home ranges.

### Burrowing patterns

The architecture of burrow systems of subterranean rodents is assumed to be affected by food availability and/or soil conditions [6, 7, 48]. Our findings that burrow system parameters of *S*. *galili* were linked to habitat differences in ecological conditions (Fig 4) corroborate this assumption. We suppose that larger, longer and more linear burrow systems in rendzina probably reflect generally lower food supply therein. The tendency for larger and longer burrow systems could be also facilitated by decreased expense of digging in softer soil (see above) and lower population density.

Analogical inter-soil differences in burrowing patterns as in our study, i.e. smaller and shorter burrow systems in soil with more food available, were observed in *S*. *carmeli* living under contrasting edaphic conditions at Mount Carmel [49]. These burrow systems were, however, much shorter than those of *S*. *galili* (40±18 m and 120±80 m, respectively). We suggest that the inter-soil differences in burrow system parameters found in both studies are due to relatively low productivity in rendzina soils and relatively high productivity of both terra rossa and basaltic soil. The effect of the difference could be further reinforced by physical workability of the soils. Generally smaller burrow systems of *S*. *carmeli* may also reflect food supply and/or soil conditions. Unfortunately, no quantitative analyses of those factors are available for that study.

Spalacines are very aggressive [50] and forcible territory takeovers ending sometimes with the death of their original holders occurs in this group [51]. This could be an important factor affecting burrowing pattern especially if animals live in close contact as in basaltic soil at our study microsite (see Fig 1). Under such conditions mole rats can either directly burrow into “no man’s land” or increase the complexity of burrows within the existing territory. Indeed, burrow systems of three neighbouring males from basaltic soil depicted in Fig 3 probably represent the latter case. Burrow systems of smaller males M843 and M737 were likely spatially limited by the activity of the large male M379 and a more reticulated pattern of those burrows may reflect their tendency to utilise limited space more thoroughly. Higher branching of burrow systems was found also at higher population densities in geomyids [27, 52].

### Population structure

Population densities of *S*. *galili* were 10 and 2 individuals per ha in basaltic soil and rendzina, respectively. These values are comparable with population densities in other spalacines (0.1–20 individuals per ha, reviewed by Savić & Nevo [11]) and some other subterranean rodents, e.g. *H*. *argenteocinereus* (4.6–5.2; [23, 48]). Fossorial rodents which consume a larger proportion of aboveground vegetation usually live under higher population densities than their strictly subterranean counterparts, reaching more than 100 individuals per ha in some ctenomyids and geomyids [27, 53]. In Israel, overall population density is 1.4, 1.8, 1.0 and 0.9 individuals per ha for *S*. *galili*, *S*. *golani*, *S*. *carmeli* and *S*. *judaei*, respectively, and it generally decreases with increasing aridity of a given habitat [54]. Comparable estimates to those derived from our study are available for *S*. *carmeli* living in rendzina (2.5 individuals per 1000 m^2^) and terra-rossa soils (7.7 individuals per 1000 m^2^) at Mount Carmel [49]. Although the relative difference in the mole rat population densities between the soils was practically identical to that of the present study, the absolute densities were almost one order higher than in our study. Such extremely high population density is surprising and it might be, for example, caused by factors such as an unusually abundant food supply and/or *Sarcopoterium* shrubs missing in those habitats.

Populations of subterranean rodents are generally at equilibrium, with population density close to the carrying capacity of a particular habitat [55]. These populations usually contain a high proportion of adults, mainly due to the high mortality of subadults during dispersal [44]. The smallest individuals in our sample (80–100 g) were probably individuals born during the previous reproductive season (i.e. less than one year old). As Israeli spalacines probably have the potential to reproduce in their first winter [56], we can consider all captured individuals of both population of *S*. *galili* sexually mature. The majority of parturitions in the study population of *S*. *galili* likely occur from February to March (see also [42] for details on reproduction of *S*. *carmeli*). If the gestation period is approximately one month [57], we can estimate that the mating season of *S*. *galili* is roughly in December—January, similarly to *S*. *carmeli* [42]. Young mole rats start constructing their own burrows from the age of eight weeks when they still rely on the maternal food supply and they become fully independent after 12 weeks [24]. Thus, in *S*. *galili*, dispersal likely takes place during April and May.

The histograms of body masses (Fig 5) revealed interesting differences between sexes. Whereas in females the histogram has a Gaussian shape, in males it was rather platykurtic with no clear peak. This dissimilarity can be attributed to male competition, as males may benefit from large body mass due to increased fighting ability, whereas females optimize their investment into their growth and reproduction.

Although sex ratios in both populations of *S*. *galili* did not diverge from parity, a more female-biased ratio was found in basaltic soil compared with rendzina (male-to-female ratio: 1:1.6 and 1:1.1 for basaltic soil and rendzina, respectively). A female-biased sex ratio is usually attributed to the male mating strategy in subterranean rodents [23, 27, 58], in which aggressive interactions and/or aboveground searches for mates may lead to higher male mortality. In subterranean rodents, aggressive interactions occur mainly when population density is high [44]. Our findings support this hypothesis, because in the microsite with higher population density (basaltic soil), the sex ratio was more female-biased.

### Implications for possible sympatric speciation

Two recent studies have brought genetic evidence which is likely ample enough to differentiate the rendzina and basaltic soil populations of *S*. *galili* at the study microsite (based on amplified fragment length polymorphism markers [13] and both markers and full sequence of the mtDNA [16]). In the present study, we show that differences between ecological conditions in the two soil types affect burrowing patterns and various population characteristics of *S*. *galili* in a predictable way. Discontinuity between resources and/or habitats is among the most important ecological feature facilitating sympatric speciation [59], which makes the idea of ongoing incipient sympatric speciation at the microsite feasible. Nevertheless, sympatric speciation would likely work with adaptive traits which are advantageous in one and disadvantageous in the other of the abutting environments. In this context, some behavioural traits, such as burrowing patterns, are more likely to be flexible in respect to seasonally varying ecological conditions. We therefore propose that the adaptive traits playing an important role in the supposed sympatric speciation of blind mole rats should be examined within physiological, morphological, and life-history traits that would be incapable of pronounced flexibility or seasonal acclimation.
